# Functional gene arrays-based analysis of fecal microbiomes in patients with liver cirrhosis

**DOI:** 10.1186/1471-2164-15-753

**Published:** 2014-09-02

**Authors:** Yanfei Chen, Nan Qin, Jing Guo, Guirong Qian, Daiqiong Fang, Ding Shi, Min Xu, Fengling Yang, Zhili He, Joy D Van Nostrand, Tong Yuan, Ye Deng, Jizhong Zhou, Lanjuan Li

**Affiliations:** State Key Laboratory for Diagnosis and Treatment of Infectious Disease, Collaborative Innovation Center for Diagnosis and Treatment of Infectious Diseases, The First Affiliated Hospital, Zhejiang University, Hangzhou, 310003 PR China; Institute for Environmental Genomics, Department of Microbiology and Plant Biology, University of Oklahoma, Norman, OK 73019 USA; State Key Joint Laboratory of Environment Simulation and Pollution Control, School of Environment, Tsinghua University, Beijing, 100084 China; Earth Sciences Division, Lawrence Berkeley National Laboratory, Berkeley, CA 94720 USA

**Keywords:** End-stage liver disease, Intestines, Microbial communities, Alcohol, Microarray

## Abstract

**Background:**

Human gut microbiota plays an important role in the pathogenesis of cirrhosis complications. Although the phylogenetic diversity of intestinal microbiota in patients with liver cirrhosis has been examined in several studies, little is known about their functional composition and structure.

**Results:**

To characterize the functional gene diversity of the gut microbiome in cirrhotic patients, we recruited a total of 42 individuals, 12 alcoholic cirrhosis patients, 18 hepatitis B virus (HBV)-related cirrhosis patients, and 12 normal controls. We determined the functional structure of these samples using a specific functional gene array, which is a combination of GeoChip for monitoring biogeochemical processes and HuMiChip specifically designed for analyzing human microbiomes. Our experimental data showed that the microbial community functional composition and structure were dramatically distinctive in the alcoholic cirrhosis. Various microbial functional genes involved in organic remediation, stress response, antibiotic resistance, metal resistance, and virulence were highly enriched in the alcoholic cirrhosis group compared to the control group and HBV-related cirrhosis group. Cirrhosis may have distinct influences on metabolic potential of fecal microbial communities. The abundance of functional genes relevant to nutrient metabolism, including amino acid metabolism, lipid metabolism, nucleotide metabolism, and isoprenoid biosynthesis, were significantly decreased in both alcoholic cirrhosis group and HBV-related cirrhosis group. Significant correlations were observed between functional gene abundances and Child-Pugh scores, such as those encoding aspartate-ammonia ligase, transaldolase, adenylosuccinate synthetase and IMP dehydrogenase.

**Conclusions:**

Functional gene array was utilized to study the gut microbiome in alcoholic and HBV-related cirrhosis patients and controls in this study. Our array data indicated that the functional composition of fecal microbiomes was heavily influenced by cirrhosis, especially by alcoholic cirrhosis. This study provides new insights into the functional potentials and activity of gut microbiota in cirrhotic patients with different etiologies.

**Electronic supplementary material:**

The online version of this article (doi:10.1186/1471-2164-15-753) contains supplementary material, which is available to authorized users.

## Background

The gastrointestinal tract harbors a complex and diverse microbial community, which plays important roles in host nutrition, immune function, health and disease. Increasing evidence suggests that dysbiosis of intestinal microbiota plays important roles in the pathogenesis of complications of cirrhosis, such as spontaneous bacterial peritonitis, and hepatic encephalopathy [1]. Bacterial translocation has been postulated as the main mechanism [2]. And intestinal bacterial overgrowth is one of the most important factors that facilitate bacterial translocation in cirrhosis [3]. Most recently, culture-independent methods including real-time PCR and high-throughput sequencing were used to characterize the structure of fecal microbiota in cirrhotic patients. Compositional changes have been linked with cirrhosis, including a significant increase of *Proteobacteria* and *Fusobacteria*, and a corresponding decrease of *Bacteroidetes* in cirrhotic patients using 454 sequencing of 16S rDNA gene [4]. Abnormal fecal microbiota functions in patients with hepatitis B virus (HBV) liver cirrhosis has also been revealed using shotgun metagenomic sequencing [5].

Metagenomic pyrosequencing of intestinal microbiota have led to the discovery of novel genes from uncultivated microorganisms, assembly of whole genomes from community DNA sequence data and comparison of microbial community composition under different physical conditions [6]. The human gut microbiome has significantly enriched metabolism of carbohydrates, amino acids, and xenobiotics, and biosynthesis of vitamins and isoprenoids, when compared with the average content of previous sequenced microbial genome [7]. There are a wide array of shared microbial genes among sampled individuals, comprising an extensive, identifiable ‘core microbiome’ at the gene level [8]. Several studies have described changes in the gene content of the gut microbiome across health and disease [9–11].

Functional gene arrays (FGAs) are the microarrays containing probes which target genes involved in a variety of functional processes and are powerful high throughput tools for monitoring the physiological status and functional activities of microbial populations and communities [12–14]. Different types of FGAs have been developed for analyzing microbial functional community structure [15], such as, GeoChip 4.0, which contains about 82,000 probes and targets about 142,000 genes from 410 functional gene families involved in nitrogen, carbon, sulfur and phosphorus cycling, antibiotic resistance, virulence factors and bacterial phage-mediated lysis (Additional file 1: Table S1), and HuMiChip 1.0, which contains 36,082 probes targeting ~50,000 gene coding DNA sequences from 139 key functional genes in various metabolic pathways (e.g., the metabolism of amino acids, carbohydrates, energy, lipids, glycan, cofactors, vitamins, and nucleotides) (Additional file 2: Table S2). Since human microbiomes have many general functions which GeoChip targets, combining these two types of FGAs would be very powerful in dissecting the functional composition and structure of human microbiomes.

We hypothesized that gut dysbiosis in cirrhosis would be related to altered microbial functional structure and that the etiology of cirrhosis would further affect the gut microbiome. Alcoholic and HBV are the main causes of cirrhosis in Western country and China, respectively. In this study, we characterized the functional structure of fecal microbial communities in patients with alcoholic cirrhosis and HBV-related cirrhosis compared to healthy controls using GeoChip 4.0 + HuMiChip 1.0. We focused on the variations of fecal microbiomes in relation to (i) the presence of cirrhosis and (ii) the etiology of cirrhosis, for example, chronic alcohol consumption. Our results indicated that cirrhosis, especially alcoholic cirrhosis, has a tremendous impact in the functional composition of fecal microbiomes.

## Results

### Overview of functional gene diversity and structure of fecal microbial communities

**Figure 1:**
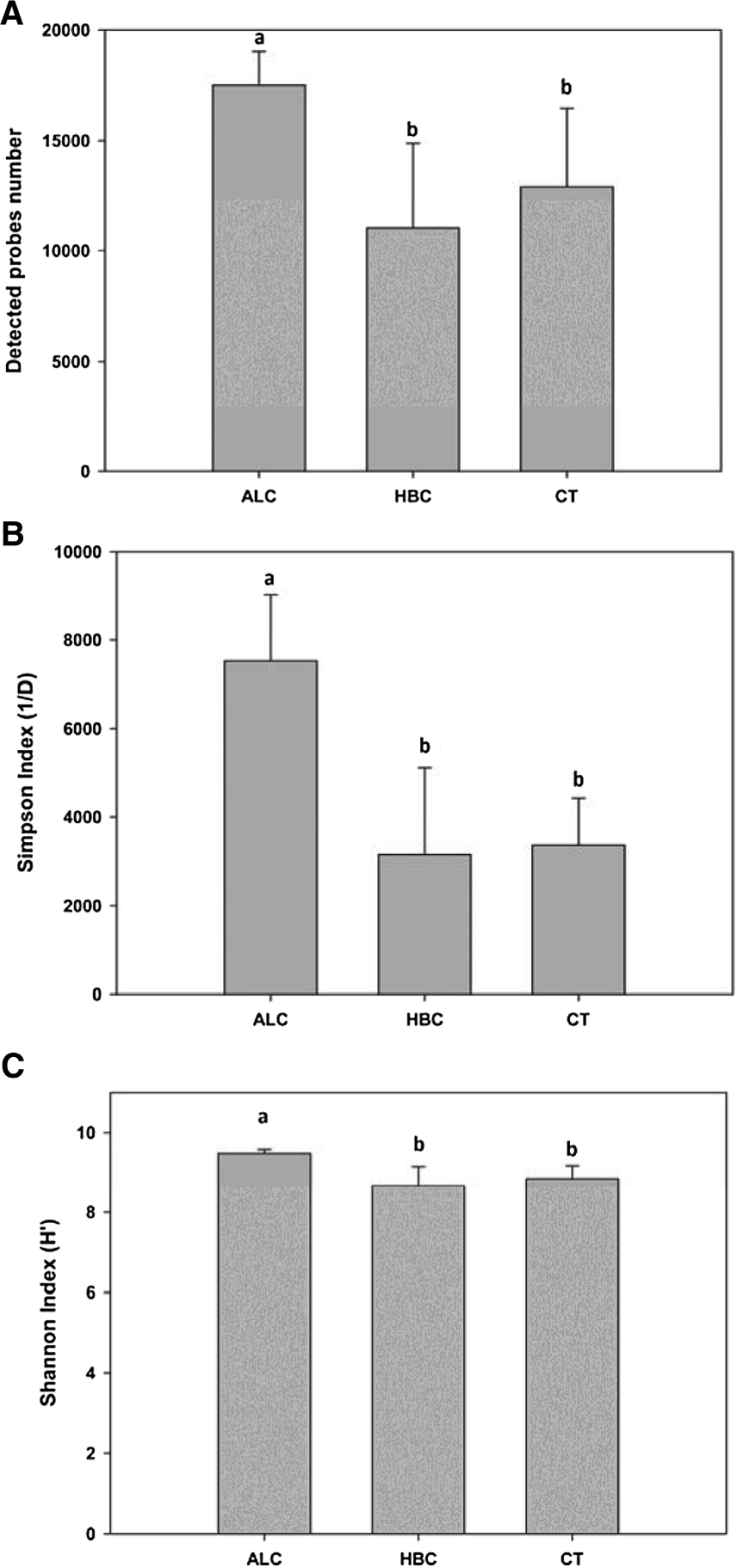
**Number of probes detected and diversities of the microbial community in alcoholic cirrhosis,** **HBV**-**related cirrhosis,** **and controls.** Gene number (Figure 1 **A**), Simpson Index (Figure 1 **B**), and Shannon Index (Figure 1 **C**) were presented as mean ± SD. Different letters above the bar (a, b) indicated statistical difference at a P value of <0.05 among groups by one-way ANOVA and Tukey’s test. HBV-related cirrhosis (HBC), healthy controls (CT), and alcoholic cirrhosis (ALC).

Based on all detected functional genes, the community diversity was assessed by richness (number of probes detected per sample), Shannon-Weiner (H’) and Simpson’s (1/D) indices (Figure 1). Overall, the number of probes detected was significantly (p < 0.01) higher in alcoholic cirrhosis (ALC) than in HBV-related cirrhosis (HBC) and controls (CT), while there was no significant difference between the HBC and CT groups. Similarly, the overall microbial functional diversity was also significantly (p < 0.001) higher in ALC than in HBC and CT based on Shannon-Weiner (H’) and Simpson’s (1/D) indices, while the difference was not significant between HBC and CT groups.

**Figure 2:**
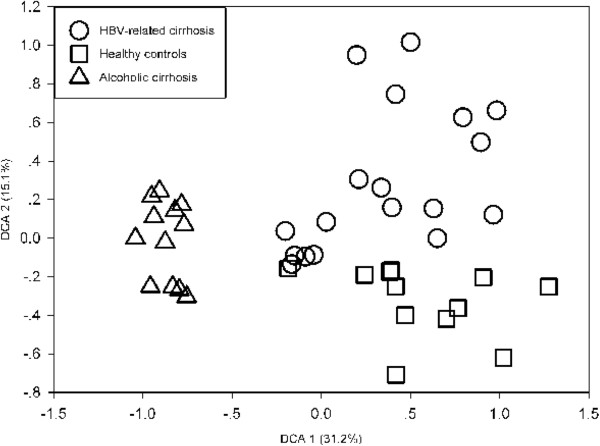
**Detrended correspondence analysis of the array data.** Circles indicate samples with HBV-related cirrhosis (HBC), squares indicate healthy controls (CT), and triangles indicate samples with alcoholic cirrhosis (ALC).

Detrended correspondence analysis (DCA) of all detected genes was used to examine overall functional structure changes in the microbial communities. Resulting ordination plots showed a marked difference among the three groups and explained 46.3% of the total variations (Figure 2). ALC samples were clearly separated from HBC and CT samples along DCA1 (31.2%), while HBC and CT were distinct along DCA2 (15.1%).

**Figure 3:**
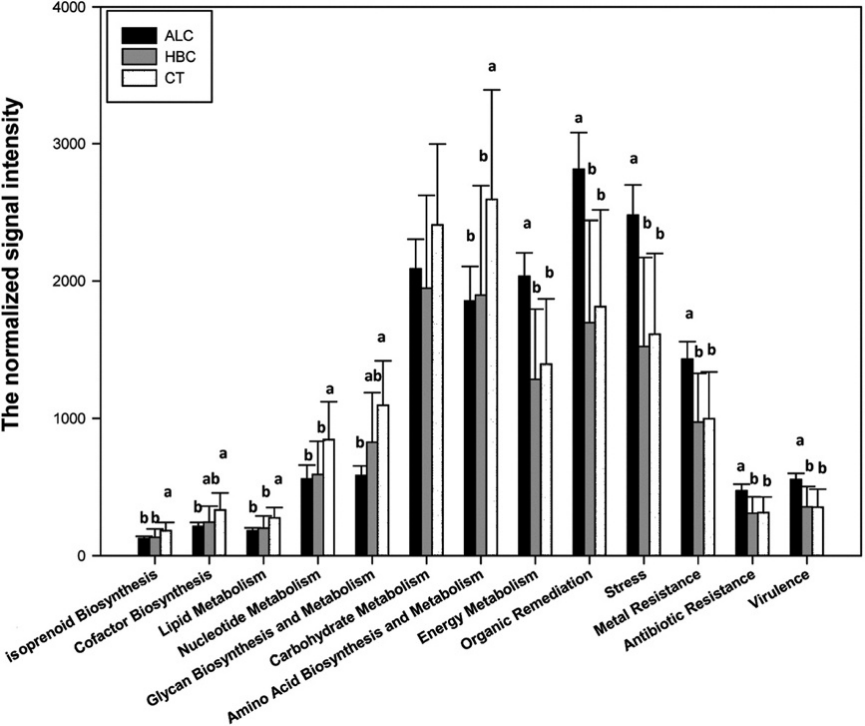
**The signal intensities of detected functional categories in alcoholic cirrhosis,** **HBV**-**related cirrhosis,** **and controls.** All data are presented as mean ± s.e. Different letters above the bar (a, b) indicate statistical difference at a P value of <0.05 among groups by one-way ANOVA and Tukey’s test. No letter was labelled if there was no significant difference observed.

The average signal intensities of functional categories were compared among the three groups (Figure 3). The functional categories involved in nutrient metabolism were significantly (p < 0.05) lower in ALC and HBC than in CT, including the functional categories of isoprenoid biosynthesis, lipid metabolism, nucleotide metabolism, and amino acid biosynthesis and metabolism. For glycan biosynthesis and metabolism and cofactor biosynthesis, the relative abundance was significantly (p < 0.05) lower in ALC than in CT, while the difference was not significant between HBC and CT group. Functional categories, such as energy metabolism, organic remediation, stress response, antibiotic resistance, metal resistance and virulence, were significantly (p ≤ 0.001) enriched in ALC compared to CT and HBC, while there was no significant difference between HBC and CT.

### Shifts of genes in response to cirrhosis

**Figure 4:**
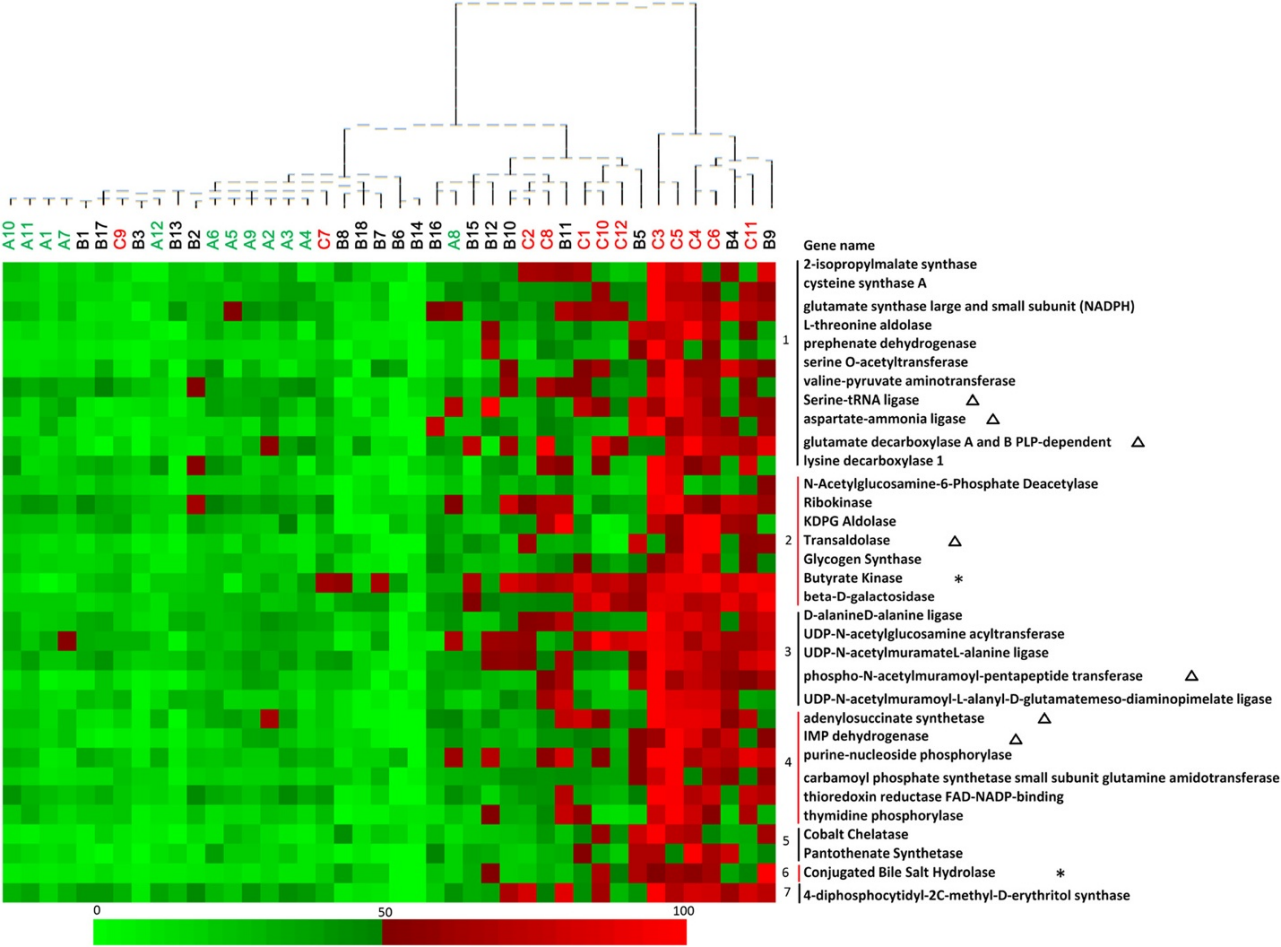
**Abundance distributions of the 33 functional genes in response to cirrhosis.** This is a heat map showing abundance distributions of the 33 functional genes in response to cirrhosis. The colume is sample, with the sample name and cluster matrix on the top of the figure. The row is the gene signal intensity, with the gene name on the right. The color intensity indicates difference in signal intensities. To show the distribution of the genes with lower abundance, the colored squares of each row were scaled to indicate the relative ratios of the genes among 42 subjects. “*” behind the gene name indicated this gene was found significantly (p < 0.05) lower in HBC than in CT, and even lower in ALC than in HBC samples. “∆” indicated the gene was correlated significantly with Child-Pugh score. The number in front of the gene names indicated the functional category which they belong to. 1. Amino acid biosynthesis and metabolism; 2. Carbohydrate metabolism; 3. Glycan biosynthesis and metabolism; 4. Nucleotide metabolism; 5. Cofactor biosynthesis; 6. Lipid metabolism; 7. Isoprenoid biosynthesis.

Cirrhosis mostly affected the abundance of functional genes involved in nutrient metabolism. In this study, we identified 33 genes, whose alteration was consistent between HBC and ALC relative to controls (Figure 4).

(i) Amino acid biosynthesis and metabolism. A total of 11 genes involved in amino acid biosynthesis and metabolism were detected significantly (p < 0.05) reduced in both cirrhosis group, including amino acid biosynthesis genes (2-isopropylmalate synthase gene, cysteine synthase A gene, NADPH gene, L-threonine aldolase gene, prephenate dehydrogenase gene, serine O-acetyltransferase gene, valine-pyruvate aminotransferase gene, serine-tRNA ligase gene, and aspartate-ammonia ligase gene), and amino acid metabolism genes (glutamate decarboxylase A and B PLP-dependent gene, and lysine decarboxylase 1 gene).
(ii) Carbohydrate metabolism. Carbohydrate metabolism is one of the most important functions the distal gut microbiome provides for the host. The abundance of genes involved in carbohydrate metabolism was significantly (p < 0.05) lower in ALC and HBC than in CT, including genes for KDPG aldolase, transaldolase, glycogen synthase, ribokinase, N-acetylglucosamine-6-phosphate deacetylase, and beta-D-galactosidase. Butyrate serves as the principal energy source for colonocytes and may fortify the intestinal mucosal barrier by stimulating their growth [16]. The genes encoding butyrate kinase, which participate in butyrate metabolism, were significantly (p < 0.001) lower in HBC than in CT, and even lower in ALC than in HBC.
(iii) Glycan biosynthesis and metabolism. The array data demonstrated 5 genes of glycan biosynthesis and metabolism significantly declined in both cirrhosis groups relative to controls, including D-alanineD-alanine ligase gene, UDP-N-acetylglucosamine acyltransferase gene, UDP-N-acetylmuramateL-alanine ligase gene, phospho-N-acetylmuramoyl-pentapeptide transferase gene, and UDP-N-acetylmuramoyl-L-alanyl-D-glutamatemeso-diaminopimelate ligase gene (p < 0.05).
(iv) Nucleotide metabolism. The abundance of genes involved in nucleotide metabolism was significantly (p < 0.05) lower in ALC and HBC than in CT, including genes for purine metabolism (adenylosuccinate synthetase gene, IMP dehydrogenase gene, and purine-nucleoside phosphorylase gene), and pyrimidine metabolism (carbamoyl phosphate synthetase small subunit glutamine amidotransferase gene, thioredoxin reductase FAD-NADP-binding gene, and thymidine phosphorylase gene).
(v) Cofactor biosynthesis and others. For cobalt chelatase gene (encoding vitamin B_12_) and pantothenate synthetase gene (encoding pantothenic acid), the signal intensities were significantly (p < 0.001) lower in ALC and HBC than in CT. The signal intensities of isoprenoid biosynthesis gene encoding 4-diphosphocytidyl-2C-methyl-D-erythritol synthase were significantly (p < 0.05) lower in ALC and HBC than in CT. Bacterial bile salt hydrolase participates in the conversion of primary bile acids to secondary bile acids [17]. The signal intensity of conjugated bile salt hydrolase gene was found significantly (p < 0.05) lower in HBC than in CT, and even lower in ALC than in HBC samples.

### Correlations between gene abundance and child-pugh scores

**Figure 5:**
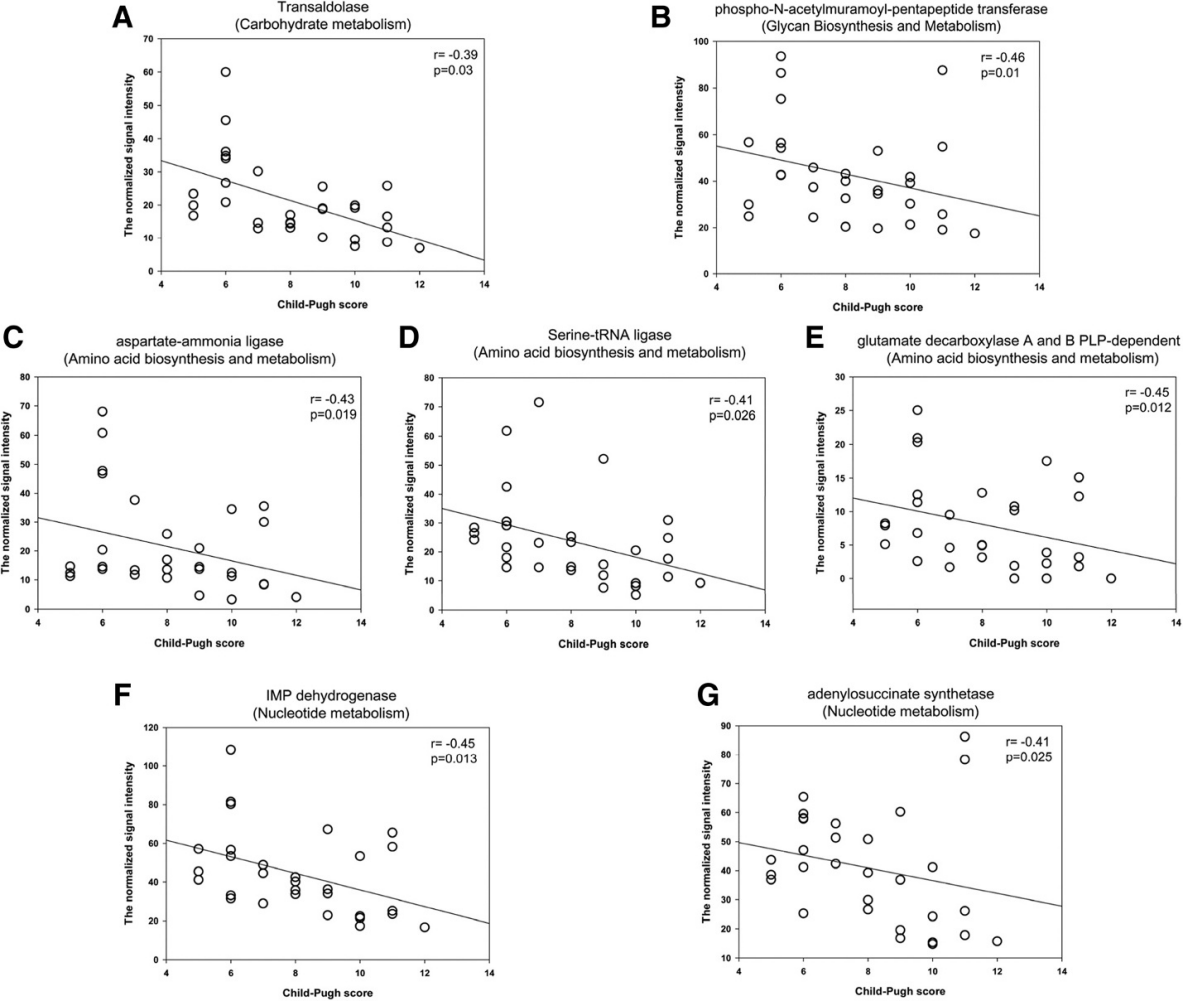
**Correlation between Child**-**Pugh score and the signal intensity of functional genes.** Seven genes correlated negatively with the severity of liver disease, as estimated by the Child-Pugh score, including genes for genes for carbohydrate metabolism (transaldolase) (Figure 5 **A**), glycan metabolism (phospho-N-acetylmuramoyl-pentapeptide transferase gene) (Figure 5 **B**), amino acids synthesis (aspartate-ammonia ligase, serine-tRNA ligase and glutamate decarboxylase A and B PLP-dependent gene) (Figure 5 **C**, **D**, and **E**), and nucleotide metabolism (IMP dehydrogenase gene and adenylosuccinate synthetase gene) (Figure 5 **F**, **G**). The gene name is on the top of each figure, with the functional category name in brackets. The Spearman Rank correlation (r) and probability (p) with Bonferroni correction was used to evaluate statistical importance.

Among those genes with decreased abundances in both cirrhosis groups, we found 7 genes negatively correlated with the severity of liver disease, as estimated by the Child-Pugh score, including aspartate-ammonia ligase gene (r = −0.427, p = 0.019), serine-tRNA ligase gene (r = −0.406, p = 0.026), and glutamate decarboxylase A and B PLP-dependent gene (r = −0.453, p = 0.012) for amino acid metabolism, transaldolase gene (r = −0.397, p = 0.03) for carbohydrate metabolism, phospho-N-acetylmuramoyl-pentapeptide transferase gene (r = −0.463, p = 0.014) for glycan metabolism, adenylosuccinate synthetase gene (r = −0.449, p = 0.013) and IMP dehydrogenase gene (r = −0.409, p = 0.025) for purine metabolism (Figure 5).

### Shifts of genes in response to alcohol

**Figure 6:**
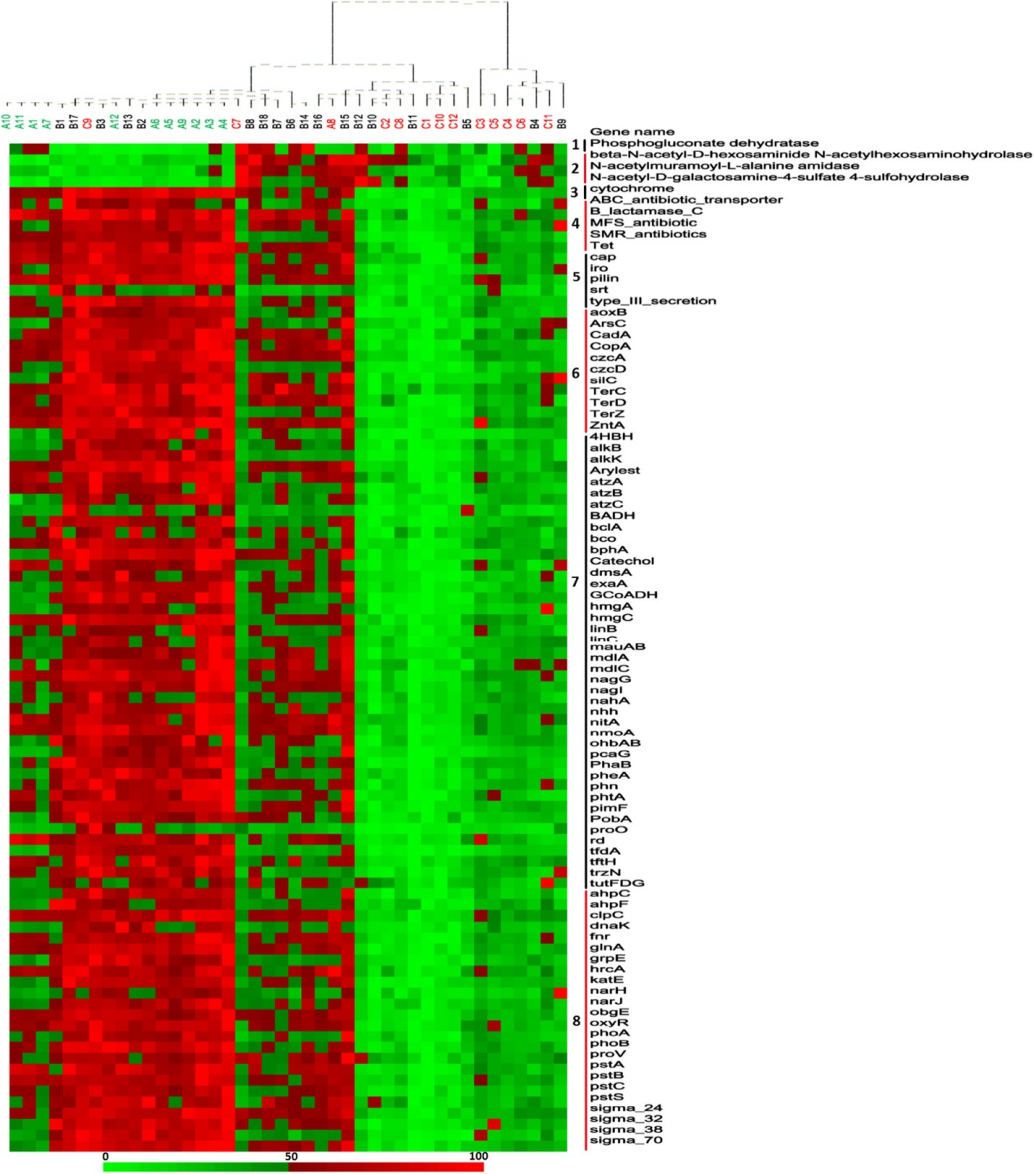
**Abundance distributions of the 92 functional genes in response to alcohol consumption.** This is a heat map showing abundance distributions of the 92 functional genes in response to cirrhosis. The column is the sample, with the sample name and cluster matrix on the top of the figure. The row is the gene signal intensity, with the gene name on the right. The colour intensity indicates difference in signal intensities. To show the distribution of the genes with lower abundance, the colored squares of each row were scaled to indicate the relative ratios of the genes among 42 subjects. The number in front of the gene names indicated the functional category which they belong to. 1. Carbohydrate metabolism; 2. Glycan biosynthesis and metabolism; 3. Energy process; 4. Antibiotic resistance; 5. Virulence; 6. Metal resistance; 7. Organic remediation; 8. Stress response.

A comparison of ALC with HBC and CT revealed that 92 genes were uniquely altered in response to alcohol consumption (Figure 6). Among them, 4 genes were found significantly (p < 0.05) reduced in ALC than in HBC and CT, including carbohydrate metabolism gene (phosphogluconate dehydratase gene) and glycan biosynthesis and metabolism gene (beta-N-acetyl-D-hexosaminide N-acetylhexosaminohydrolase gene, N-acetyl-D-galactosamine-4-sulfate 4-sulfohydrolase gene, and N-acetylmuramoyl-L-alanine amidase gene). Most of the over-represented genes were involved in functional categories of organic remediation, stress response, and antibiotic or metal resistance. The abundance of cytochrome gene in energy metabolism was found significantly increased in ALC than in HBC and CT.

(i) Organic degradation. A substantial number of genes (42) involved in degradation of organic compounds were detected with different abundances, especially those involved in degrading aromatic carboxylic acid, BTEX and related aromatics, chlorinated aromatics, herbicides and pesticides related compounds, and chlorinated solvents. Most of these genes (e.g., *pimF*, *nagG*, *nmoA*, and *bphA*, Catechol genes, GCoADH genes) showed significantly (p < 0.05) increased abundances in ALC than in CT and HBC (Additional file 1: Table S1). Phthalates within alcoholic beverages have attracted more and more attention for their health hazards in recent years. The abundance of *phtA* gene, encoding phthalate 4, 5-dioxygenase, was significantly (p < 0.001) higher in ALC than in HBC and CT.
(ii) Stress response. A total of 24 genes involved in stress response showed significantly (p < 0.05) increased abundances in ALC than in CT and HBC, including oxygen stress (*ahpC*, *ahpF*, *fnr*, *katE*, and *oxyR*), oxygen limitation (*narH*, and *narJ*), nitrogen limitation (*glnA*), heat shock (*dnaK*, *grpE*, *hrcA*), phosphate limitation (*pstA*, *pstB*, *pstC* and *pstS*), and osmotic stress (*proV*).
(iii) Virulence. Functional genes associated with bacterial virulence were found over-represented in ALC samples compared to CT and HBC. The signal intensities of the genes encoding the bacterial secretion system (type_Ш_secretion), catabolite activator protein (*cap*), iron-regulated TonB-dependent receptor (*iro*), pilin protein (*pilin*), and sortase family protein (*srt*), were significantly (p < 0.001) higher in ALC than in HBC and CT.
(iv) Antibiotic resistance. Regarding antibiotic resistance, 5 genes were detected in significantly (p < 0.05) higher abundance in ALC than other groups, including genes for ABC_antibiotic_transporter, ß-lactamase_C, MFS_antibiotic, SMR_antibiotics, and Tet genes.
(v) Metal resistance. Functional genes associated with metal resistance were found over-represented in ALC samples compared to CT and HBC. The signal intensities of the genes encoding arsenite oxidase (*aoxB*), arsenate reductase (*ArsC*), cadmium-translocating P-type ATPase (*CadA*), copper-transporting P-type ATPase (*CopA*), heavy metal efflux pump protein (*czcA*, *czcD*, *ZntA*), RND efflux system outer membrane lipoprotein (*silC*), tellurite resistence protein (*TerC*, *TerD*, *TerZ*), were found singnificantly (p < 0.05) higher in ALC than in HBC and CT.

## Discussion

Although several studies have reported composition variations between cirrhotic patients and healthy controls, little is known about bacterial gene content and their potential functions in cirrhosis of different etiologies [4, 18]. There is a growing recognition of the need to study the functional profile of fecal microbiota. Here we have presented a functional gene array-based analysis of fecal microbiota in patients with alcoholic cirrhosis and HBV-related cirrhosis.

Different methods can be used to study complex microbial populations. Recently, the development of the new-generation sequencing technologies challenged the use of DNA microarrays in microbial community studies. The main limitation of microarrays is their inability to reveal novel species in any samples, since the arrays can only detect those sequences for which they contain probes. However, there are several main advantages of microarrays, include (i) ability to profile one sample at a time, which is useful in clinical studies and as a diagnostic tool; (ii) quantitative nature of the acquired data allowing direct comparison between samples; (iii) quick sample preparation and short processing and data acquisition time (only 24 hours from sample to data using GeoChip); (iv) currently lower costs compared with shotgun sequencing; and (v) resistance to contaminants of host genome, which is useful for mucosa samples containing a large percentage of host DNA [19, 20].

Although a few samples overlapped, there is a trend of separation between cirrhosis subjects and controls. Functional categories prominent in healthy controls were mostly related to nutrient metabolism (Figure 3). Our results indicated that the metabolic potential of the gut microbiota was significantly affected, analogous to a previous study which reported a decrease in concentration of a large number of metabolites in patients with cirrhosis based on metabolomic analysis of feces samples [21]. The exact reason for reduced gut microbial metabolic potential in cirrhotic patients remains unclear. The fact of low protein diet in the majority of cirrhotic patients may play a role in it. Although not recommended, patients with cirrhosis usually took low protein diet to avoid hepatic encephalopathy [22]. The effect of diet on gut microbiome has been verified in several studies [23, 24]. A controlled-feeding study of 10 subjects showed that microbiome composition changed detectably within 24 hours of initiating a high fat/low fiber or low fat/high fiber diet [25]. In the current study, the microbiome of cirrhosis had a lower relative abundance of genes associated with the biosynthesis and metabolism of several amino acids, including leucine (2-isopropylmalate synthase gene), serine (serine-tRNA ligase gene and L-threonine aldolase gene), cysteine (serine O-acetyltransferase gene and cysteine synthase A gene), and valine (valine-pyruvate aminotransferase gene) (Figure 3). Biosysthesis of amino acids are critical for the growth of all microbes, but the amount and profile of amino acids produced, and production of other protein-containing metabolites have been reported to vary depending on dietary composition in animal studies [26, 27]. Malnutrition has increasingly been acknowledged as an important prognostic factor, which can influence the clinical outcome of cirrhotic patients [28]. Alcoholic beverages, which contribute little to the nutritional requirements of the body, are usually associated with food deficiency due to decreased appetite [29]. Alcohol ingestion has been shown to decrease vitamin B_12_ absorption in volunteers after several weeks of intake [30]. In relation to this, our data revealed that cobalt chelatase gene was significant underrepresented in cirrhosis groups, especially in alcoholic cirrhosis group. Cobalt chelatase is an important pathway in vitamin B_12_ biosynthesis [31]. Together with diet alterations, reduced metabolic potential of gut microbiome might increase the risk of malnutrition in cirrhosis.

Another class of functions changed in the cirrhosis groups was bile salt metabolism. Bile acids, drugs and toxins undergo extensive enterohepatic circulation, which is also called gut-liver axis [32]. Bile acids play a major role in several hepatic and intestinal diseases. Intestinal bacteria are known to participate in bile acid metabolism by generating secondary bile acids (deconjugation, dehydroxylation) [17]. The initial “gateway” reaction in the bacterial metabolism of conjugated bile acids is mediated by bile salt hydrolase [33]. In cirrhosis, decrease in intestinal intraluminal concentrations of bile acids have been ascribed to decrease secretion and increased deconjugation by enteric bacteria [2]. Nevertheless, our results demonstrated the abundance of conjugated bile salt hydrolase gene was significantly lower in cirrhosis than in controls. In a recent metagenomic research of patients with HBV-related cirrhosis using high-throughput sequencing, genes annotated as bile salt hydrolases were also detected in higher abundance in the normal microbiota than in the cirrhotic microbiota [5]. In particular, cirrhotic patients showed an altered fecal bile acid profile with a decreased conversion of primary to secondary fecal bile acids [34]. It is thus tempting to speculate that decrease synthesis of bile acids in the liver contributes more importantly to decrease in intestinal intraluminal concentrations of bile acids in cirrhosis.

Enzyme responsible for butyrate metabolism (butyrate kinase gene) was found in less abundant in both cirrhosis groups, especially in alcoholic cirrhosis group (Figure 4). Our results are in agreement with the 16S rRNA based analysis, which shows that many of the bacterial genera significantly less abundant in cirrhotic patients are butyrate producers and mucin degraders, such as *Faecolibacterium* and *Prevotella* [35]. Butyrate is known as the principal energy source for colonocytes and may fortify the intestinal mucosal barrier by stimulating mucin synthesis [36]. Recent endoscopic studies reveal mucosal alterations in the gastrointestinal tract including inflammatory-like changes and vascular lesions in cirrhosis [37]. Linking together these observations, a microbiota-dependent reduction of butyrate production may possibly contribute to the nutritional status of epithelial cell in the intestine in cirrhosis, and potentially lead to mucosal changes, hypertensive enteropathy and bacterial translocation indirectly.

The current results from the array analysis showed considerable variations of the community functional structure were observed in response to alcohol consumption. Higher microbial richness and genetic diversity were observed in ALC samples than in HBC or CT samples. The bacterial genomes have been shown to be highly plastic and are frequently reorganized through genetic rearrangements and gene transfer, which can help them adapt to distinct ecological conditions [38]. High level of heterogeneity and diversity among strains were observed under stressful ecosystem such as high ethanol content, low pH and temperature [39]. Alcohol ingested orally is transported to the colon by blood circulation, and due to its high water solubility, intracolonic ethanol levels are equal to those in the blood [40]. In patients with cirrhosis, extrahepatic ethanol metabolism, via microbial oxidation in the colon, is responsible for a large portion of ethanol metabolism [41]. It was recently shown that low concentrations of alcohol can enhance microbial activity in the human body. For example, in *Staphylococcus aureus*, ethanol supplementation stimulated the expression of genes involved in stress response, and biofilm formation [42]. Treatment of *S. aureus* with alcohol resulted in increased transcription of the biofilm-promoting genes *icaA* and *icaD*, as well as the antibiotic resistance gene *mmpL* (multidrug efflux pump), the efflux pump gene *mepA*, and the sensor histidine kinase gene *vraS* [43]. In relation to this, functional genes related to stress response and antibiotic resistance were significantly over-represented in our study.

Our results also revealed great metabolic potential in the alcoholic cirrhosis group for xenobiotic degradation. Numerous gut microbial genes are related to xenobiotic degradation, including non-modified and halogenated aromatic compounds [44]. More than 700 aromatic compounds have been isolated and identified from various wines, which derive from by-products of yeast fermentation and additives. Phthalates are a group of industrial chemicals that have become ubiquitous environmental contaminants because of their widespread usage and high persistence in the environment [45]. Phthalates in alcoholic beverages might be from two sources: the migration from packaging materials and the use of diethyl phthalate as a denaturing agent for alcohol [46]. The abundance of *phtA* genes, encoding phthalate 4, 5-dioxygenase, was significantly higher in ALC than in HBC or CT samples. Long-term alcohol consumption could potentially lead to the enriched genes in phthalates degradation. Moreover, our results revealed metabolic potential in alcoholic cirrhosis group for benzoate metabolism, such as *bclA* gene, *nagG* gene and *4HBH* gene (Figure 6). Benzoate could enter the tricarboxylicacid cycle, and might constitute a potential energy source [47]. Aromatic compound degradation may allow for the utilisation of a wider array of substrates that may be used for energy harvesting in alcoholic cirrhosis.

In this study, we detected several genes which were negatively correlated with Child-Pugh score (Figure 5). Our results offer the possibility of using functional genes as biomarkers to estimate the severity of liver cirrhosis. Nevertheless, obtaining cultures of microbes is essential for validating these hypotheses. Furthermore, since considerable variations of the community functional structure were observed in response to alcohol consumption, individuals with alcohol consumption with or without liver disease should also be compared to validate such links in future studies. In addition, DNA-based array analysis detects only the functional potential of microbial communities, but not necessarily the actual functional activity. To understand functional activities, RNA-based array analysis is needed in future studies.

## Conclusions

Functional gene array (GeoChip 4.0 + HuMiChip 1.0) was utilized to study the gut microbiome in alcoholic and HBV-related cirrhosis patients and controls in this study. Our array data indicated that the functional composition of fecal microbiomes was mostly influenced by alcohol consumption, and secondly by cirrhosis. Alcohol consumption caused significant enrichment of functional genes including xenobiotic metabolism and virulence, while both cirrhosis groups were markedly depleted in the functional genes involved in nutrient processing, such as amino acid metabolism, lipid metabolism and nucleotide metabolism. These results have important implications for complications of cirrhosis (e.g., malnutrition and hypertensive enteropathy). Therapeutic options aimed at adjusting the gut microbiome should be a potential treatment in cirrhosis.

## Methods

### Study population

A total of 42 individuals were recruited for this research, including 3 groups: (1) 12 subjects with alcoholic cirrhosis (ALC); (2) 18 subjects with HBV-related cirrhosis (HBC); (3) 12 subjects with normal liver function by ultrasonographic examination and biochemical profiles (CT). The three groups were matched in terms of body mass index, gender, and age.

Cirrhosis was diagnosed histologically in 17 out of 30 (57%) patients and on clinical and radiological grounds in the remaining 13 (43%) patients for whom biopsy was contraindicated by uncontrolled coagulapathy and/or uncontrolled ascites. A diagnosis of alcoholic cirrhosis was established if alcohol intake had been in excess of 80 g/day in men and 30 g/day in women for more than 5 years and if testing for viral, metabolic, and immune etiologies was negative. The HBV-related cirrhotic patients and healthy controls had no history of alcohol abuse. Subjects with HBV-related cirrhosis had HBV-DNA detectable by PCR, and an absence of other (viral, drug/toxin, autoimmune, or metabolic) causes of liver disease. To avoid the influence of antibiotics or probiotics, exclusion criteria included a history of antibiotics/probiotics (or prebiotics) treatment within the previous 8 weeks of sample collection. Clinical characteristics of all groups are summarized in Table 1. Detailed characteristics of patients (e.g., hepatic encephalopathy and ascites) are shown in Additional file 3: Table S3.

**Table 1:** **Characteristics of study groups**

	Alcoholic cirrhosis	HBV-related cirrhosis	Healthy controls
	(n = 12)	(n = 18)	(n = 12)
Age	52.7 ± 13.0	48.2 ± 11.5	45.3 ± 8.4
Gerder (M/F)	9/3	12/6	8/4
BMI	20.3 ± 2.9	21.1 ± 2.3	20.6 ± 1.3
Alb	36.8 ± 6.3	32.8 ± 5.1	48.1 ± 2.5
INR	1.3 ± 0.2	1.4 ± 0.4	1.1 ± 0.1
TB	55.0 ± 33.9	63.4 ± 56.4	16.7 ± 5.1
Crea	70.0 ± 15.2	76.6 ± 16.6	67.1 ± 11.9
CP	7.7 ± 2.1	8.8 ± 2.0	None

Note. 1. Age, BMI, CP, Alb, INR, TB, and Crea were expressed as mean ± SD. 2. Measure unit: Age (yr); BMI (kg/m2); TB (umol/L); PT (S); Alb (g/L); Crea (umol/L).

Abbreviations: *BMI* body mass index, *CP* Child-Pugh score, *TB* total bilirubin, *PT* prothrombin time, Alb, serum albumin, *INR* international normalized ratio, Crea, serum creatine.

All subjects in this study provided their informed consent in writing. The use of these subjects conformed to the ethical guidelines of the 1975 Declaration of Helsinki, and was approved by the First Affiliated Hospital of Zhejiang University Institutional Review Broad.

### Sampling and DNA extraction

All fecal samples were immediately frozen on collection and stored at −70°C before analysis. Fecal DNA was extracted with the QIAamp DNA Stool Mini Kit (Qiagen, Valencia, CA, USA) as described previously [4]. DNA quality was assessed with a ND-1000 spectrophotometer (Nanodrop Inc., Wilmington, DE, USA). DNA concentrations were quantified with Quant-It PicoGreen Kit (Invitrogen Carlsbad, CA, USA) and 2 μg DNA was used for functional gene array hybridization.

### Array analysis

Details for labeling, hybridization, image processing and data processing were described previously [48, 49]. Probes that were detected in less than 10% of each group were removed.

### Statistical analysis

All hybridization data is available at the Institute for Environmental Genomics, University of Oklahoma (http://ieg.ou.edu/). Detrended correspondence analysis (DCA) was performed by the vegan package in R 2.9.1 (R Development Core Team 2006). Correlation between variables was computed by Spearman Rank correlation. To minimize false significant correlations, results were adjusted for multiple testing within each category using the Bonferroni correction. One way ANOVA with Tukey’s test was used to evaluate the difference in functional categories among the three groups. The one way ANOVA and Spearman Rank correlation were conducted in SPSS version 11.0 for Windows (SPSS Inc., Chicago, IL, USA).

## Electronic supplementary material

Additional file 1: Table S1: Summary of probe and covered coding sequence information of GeoChip 4 based on gene categories. (DOC 42 KB)

Additional file 2: Table S2: Summary of probes and covered coding sequence information of HuMiChip 1.0 based on gene categories. (DOC 38 KB)

Additional file 3: Table S3: Clinical Information Summary of Patients in Study. (DOC 77 KB)

## Abbreviations

HBV: Hepatitis B virus
DCA: Detrended correspondence analysis.
