# Supplementary material for: Functional gene arrays-based analysis of fecal microbiomes in patients with liver cirrhosis
Source: BMC Genomics. 2014 Sep 2;15(1):753. doi: 10.1186/1471-2164-15-753 (PMC4171554; doi:10.1186/1471-2164-15-753)
Supplement: Supplementary file 1 — Additional file 1: Table S1: Summary of probe and covered coding sequence information of GeoChip 4 based on gene categories. (DOC 42 KB) [file 12864_2014_6461_MOESM1_ESM.doc]

**Table S1**.Summary of probe and covered coding sequence information of GeoChip 4 based on gene categories.

| **Gene category** | **#genes or enzymes** | **#probes** | **#sequence-specific probes** | **#group-specific probes** | **#covered CDS** |
| --- | --- | --- | --- | --- | --- |
| Carbon cycling | 41 | 11,034 | 3,204 | 7,830 | 18,071 |
| Nitrogen cycling | 17 | 7,386 | 3,090 | 4,296 | 10,744 |
| Phosphorus utilization | 3 | 1,341 | 351 | 990 | 2,261 |
| Sulphur | 6 | 3,113 | 1,784 | 1,329 | 4,049 |
| Energy process | 4 | 853 | 436 | 417 | 1,131 |
| Metal resistance | 44 | 9,272 | 1,295 | 7,977 | 17,198 |
| Organic remediation | 184 | 17,056 | 4,692 | 12,364 | 28,716 |
| Antibiotic resistance | 11 | 3,334 | 589 | 2,745 | 5,533 |
| Stress | 45 | 21,541 | 1,313 | 20,228 | 40,635 |
| Bacteria phage | 40 | 1,071 | 195 | 876 | 1,987 |
| Virulence | 13 | 3,726 | 315 | 3,411 | 7,444 |
| Other | 2 | 2,245 | 822 | 1,423 | 4,042 |
| **Total** | **410** | **81,972** | **18,086** | **63,886** | **141,811** |
