# Supplementary material for: Functional gene arrays-based analysis of fecal microbiomes in patients with liver cirrhosis
Source: BMC Genomics. 2014 Sep 2;15(1):753. doi: 10.1186/1471-2164-15-753 (PMC4171554; doi:10.1186/1471-2164-15-753)
Supplement: Supplementary file 2 — Additional file 2: Table S2: Summary of probes and covered coding sequence information of HuMiChip 1.0 based on gene categories. (DOC 38 KB) [file 12864_2014_6461_MOESM2_ESM.doc]

**Table S2**.Summary of probes and covered coding sequence information of HuMiChip 1.0 based on gene categories.

| **Gene category** | **#genes** | **#probes** | **#sequence-specific probes** | **#group-specific probes** | **#covered CDS** |
| --- | --- | --- | --- | --- | --- |
| Amino acid biosynthesis and metabolism | 82 | 21,392 | 14,633 | 6,759 | 29,444 |
| Carbohydrate metabolism | 35 | 9,386 | 6,236 | 3,150 | 12,109 |
| Energy metabolism | 14 | 4,992 | 3,292 | 1,700 | 6,359 |
| Glycan biosynthesis and metabolism | 14 | 6,507 | 4,379 | 2,128 | 7,911 |
| Lipid metabolism | 6 | 2,415 | 1,585 | 830 | 2,905 |
| Cofactor metabolism | 17 | 3,660 | 2,464 | 1,196 | 4,879 |
| Isoprenoid biosynthesis | 5 | 1,841 | 1,247 | 594 | 2,517 |
| Nucleotide metabolism | 13 | 4,437 | 3,013 | 1,424 | 6,421 |
| Translation | 3 | 429 | 270 | 159 | 765 |
| **Total*** | **139** | **36,802** | **25,003** | **11,799** | **50,007** |

*Gene families targeting human microbiomes are selected from KEGG pathway database, and may participate in multiple pathways. The total number of probes and covered coding sequences is based on non-redundant genes included in all pathways, but it is not calculated as the sum of all sub-categories.
