# Supplementary material for: Functional gene arrays-based analysis of fecal microbiomes in patients with liver cirrhosis
Source: BMC Genomics. 2014 Sep 2;15(1):753. doi: 10.1186/1471-2164-15-753 (PMC4171554; doi:10.1186/1471-2164-15-753)
Supplement: Supplementary file 3 — Additional file 3: Table S3: Clinical Information Summary of Patients in Study. (DOC 77 KB) [file 12864_2014_6461_MOESM3_ESM.doc]

| Table S3. Clinical Information Summary of Patients in Study. | | | | | | | | | | | | | | |
| --- | --- | --- | --- | --- | --- | --- | --- | --- | --- | --- | --- | --- | --- | --- |
| ID | Gender | Age | BMI | Etiology | CP | HE | Ascites | Alb | TB | PT | INR | Crea | Antibiotics | Abstinent |
| A1 | M | 71 | 21.06 | Alcohol | 8 | None | None | 35.8 | 87 | 14.7 | 1.3056 | 95 | Ampicillin sulbactam | 2 months |
| A2 | M | 63 | 15.86 | Alcohol | 13 | Grade 2 | Mild | 26.4 | 65 | 17.6 | 1.5606 | 90 | None | 1 months |
| A3 | M | 32 | 19.57 | Alcohol | 7 | None | None | 34.2 | 36.2 | 12.1 | 1.071 | 63 | None | 2 months |
| A4 | F | 49 | 20.81 | Alcohol | 8 | None | Mild | 42.5 | 119.2 | 12.5 | 1.1118 | 60 | None | 2 months |
| A5 | M | 42 | 19.27 | Alcohol | 9 | None | None | 30.3 | 107.3 | 17 | 1.5198 | 53 | None | 1 months |
| A6 | M | 53 | 22.50 | Alcohol | 8 | None | None | 40.4 | 45.8 | 17.6 | 1.581 | 67 | None | 2 months |
| A7 | M | 37 | 16.78 | Alcohol | 5 | None | None | 43.5 | 23.7 | 12.2 | 1.0812 | 71 | Cefuroxime | 6 months |
| A8 | F | 58 | 24.58 | Alcohol | 7 | None | Moderate | 44.5 | 39.6 | 11.8 | 1.0506 | 82 | None | 18 months |
| A9 | M | 65 | 18.81 | Alcohol | 7 | None | None | 43.5 | 22 | 17.8 | 1.5504 | 77 | None | 6 months |
| A10 | M | 44 | 24.44 | Alcohol | 5 | None | None | 37.9 | 23.1 | 12.9 | 1.1424 | 61 | Moxifloxacin | 1 months |
| A11 | M | 71 | 17.05 | Alcohol | 6 | None | None | 28.6 | 26.7 | 14.3 | 1.275 | 78 | None | 1 months |
| A12 | F | 47 | 22.50 | Alcohol | 9 | None | None | 33.6 | 63.9 | 17.2 | 1.5198 | 43 | None | 6 months |
| B1 | M | 57 | 25.52 | HBV | 11 | None | Mild | 32.5 | 149 | 20.4 | 1.6005 | 92 | None | N |
| B2 | F | 53 | 20.39 | HBV | 9 | None | Mild | 30.5 | 36 | 17.2 | 1.4626 | 113 | None | N |
| B3 | M | 67 | 17.31 | HBV | 8 | None | Mild | 35.2 | 42 | 14.4 | 1.2125 | 84 | None | N |
| B4 | M | 26 | 19.33 | HBV | 10 | None | None | 24.4 | 152 | 16.4 | 1.3871 | 71 | Levofloxacin | N |
| B5 | M | 41 | 19.92 | HBV | 11 | None | Mild | 28.2 | 69 | 18.6 | 1.5714 | 73 | None | N |
| B6 | F | 33 | 22.05 | HBV | 12 | None | Mild | 25.4 | 56 | 22.4 | 1.8915 | 71 | None | N |
| B7 | M | 42 | 22.88 | HBV | 7 | None | None | 35.1 | 12.9 | 16.1 | 1.358 | 77 | None | N |
| B8 | M | 44 | 22.52 | HBV | 6 | None | None | 39.5 | 27 | 14.6 | 1.2319 | 69 | None | N |
| B9 | F | 62 | 22.34 | HBV | 10 | None | None | 30.2 | 128 | 25 | 2.037 | 51 | None | N |
| B10 | F | 56 | 24.08 | HBV | 9 | Grade 1 | None | 33.5 | 31 | 18.4 | 1.6393 | 49 | Levofloxacin | N |
| B11 | M | 56 | 22.61 | HBV | 6 | None | None | 31.8 | 14 | 12.3 | 1.0379 | 67 | None | N |
| B12 | M | 44 | 16.56 | HBV | 9 | None | Mild | 31.3 | 51 | 16.8 | 1.4162 | 99 | None | N |
| B13 | F | 44 | 20.59 | HBV | 7 | None | Mild | 29.9 | 9 | 12.1 | 1.0185 | 76 | Cefuroxime | N |
| B14 | M | 42 | 20.77 | HBV | 11 | None | Severe | 36.3 | 195 | 28.2 | 2.231 | 78 | None | N |
| B15 | M | 36 | 20.59 | HBV | 10 | None | Moderate | 33.3 | 43 | 15.8 | 1.3289 | 67 | None | N |
| B16 | M | 41 | 18.49 | HBV | 10 | None | Mild | 27.9 | 92 | 16.4 | 1.3871 | 81 | None | N |
| B17 | F | 63 | 23.11 | HBV | 6 | None | Mild | 41.9 | 11 | 11.3 | 0.9506 | 60 | None | N |
| B18 | M | 60 | 21.41 | HBV | 6 | None | Mild | 42.8 | 23 | 11.8 | 0.9991 | 100 | None | N |
| Note. 1. The severity of hepatic encephalopathy is graded with the West Haven Criteria. 2. Measure unit: TB (umol/L); PT (S); Alb (g/L); Crea (umol/L). 3. No patients used antibiotics in 8 weeks before sample collection. The record of antibiotic use was the history of antibiotics in the past 1 year. 4. No alcohol abuse was found in HBV -related cirrhosis. | | | | | | | | | | | | | | |
| Abbreviations: BMI, body mass index; CP score, Child-Pugh score; HE, hepatic encephalopathy; TB, total bilirubin; PT, prothrombin time; Alb, serum albumin; INR, international normalized ratio; Crea, serum creatine. | | | | | | | | | | | | | | |
